# Efficient organic mulch thickness for soil and water conservation in urban areas

**DOI:** 10.1038/s41598-021-85343-x

**Published:** 2021-03-18

**Authors:** Bing Wang, Jianzhi Niu, Ronny Berndtsson, Linus Zhang, Xiongwen Chen, Xiang Li, Zhijun Zhu

**Affiliations:** 1 School of Soil and Water Conservation, Beijing Forestry University, Beijing, 100083 China; 2 Division of Water Resources Engineering, Lund University, Box 118, 221 00 Lund, SE Sweden; 3 Centre for Advanced Middle Eastern Studies, Lund University, Box 201, 221 00 Lund, SE Sweden; 4 Department of Biological and Environmental Sciences, Alabama A&M University, Normal, AL 35762 USA; 5 China National Forestry Economic Development Research Center, National Forestry and Grassland Administration, Beijing, China; 6 Quzhou Municipal Water Resources Bureau, Quzhou, China

**Keywords:** Environmental sciences, Hydrology, Planetary science, Solid Earth sciences

## Abstract

The use of organic mulch is important for urban green applications. For urban areas in arid and semiarid regions receiving short high-intensive rainfall, rainfall characteristics, and soil slope play an important role for mulch functioning. These properties of mulch were studied. For this purpose, rainfall simulation experiments using organic mulching were conducted in Jiufeng National Forestry Park to analyze the influence of organic mulch under different slope and heavy rainfall events. The results showed that soil water content displayed a decreasing tendency with increasing mulch application. Compared to bare soil, a mulch application of 0.25 kg/m^2^ and 0.50 kg/m^2^ led to maximum soil water content and maximum runoff decrease occurred for 0.50 kg/m^2^ mulch. Higher application rate of mulch displayed less soil water content and greater runoff. The runoff amount and runoff generation rate decreased by 28–83% and 21–83%, respectively, as compared to bare soil. With a mulch application of 0.25–1.00 kg/m^2^, soil drainage accounted for 56–60% of total rainfall. Overall, an efficient mulch application was found to be 0.25–0.50 kg/m^2^. The results of this study are relevant for arid and semiarid urban regions that experience heavy rainfall.

## Introduction

Organic mulch treatment is a common method applied in landscaping, forestry, and agriculture. Urban environmental modification using mulch can be traced back to United States landscape architecture in the 1980s^1^. Today it is widely used in design of parks, green city areas, and residential communities. The urban green demand for organic mulch is growing at rate of 10% annually, and the annual volume of colored organic mulch used in United States was more than one billion cubic meter in 2016 (http://www.huamu0101.com/news/news24528.shtml). Organic mulch is defined as organic material from waste of crops, wood, paper, and animal waste that can be used as surface material on top of the bare soil. Previous studies have shown that mulch covering the soil as compared to soil mixed with mulch is more advantageous for water conservation^2^. Many studies on organic mulch have demonstrated its positive effects including amelioration of soil fertility^3,4^, reduction of soil and wind erosion^5^, soil water conservation by reducing evaporation^6^, and improvement of crop and orchard yields^7,8^. In addition, organic mulch helps to moderate temperature and protect soil and roots from extreme temperature^9,10^, prevent pest, reduce the impact of weed, and positively affect the surrounding microclimate^11^. Researchers have carried out comparative studies on effects of organic mulch on soil and water conservation using different mulch material^12^, different time scale^13^, and different soil depth^14^. These studies show that mulch addition to the soil changes its hydraulic functioning as well as its runoff characteristics. As well, runoff generation and flood mitigation depend on the efficiency of mulch addition.

Most previous research has focused on the function of organic mulch in agriculture (such as cropping yield, disease pest control, crop microclimate environment, soil water content, and plant production). A comparatively new research area is effects of organic mulch in built-up areas and urban green spaces due to that organic mulch protects the soil and is aesthetically pleasing, brings less need for pesticides and fertilizers, and improves sustainability^15^. The rapid urbanization process, e.g., in China, has led to increasing needs for organic mulch and guidelines for its use. Urbanization is often accompanied by environmental problems, such as extreme precipitation, which indirectly increases the risk of urban flood disasters^16,17^. Soil degradation in India is partly caused by flooding^12^, and cities in China face flooding problems during heavy summer rainfall^18^. Torrential rainfall in 2012 in Beijing and 2010 in Guangzhou contributed to irretrievable losses of urban ecology and people’s lives^19^. In addition, urbanization causes decrease of ecosystem services^20^, pollution and decreasing air quality^21^, and land degradation^22,23^. Greater use of organic mulch in urban development processes can decrease effects of urban dust sources, improve water and soil conditions, and guarantee the aesthetics of urban landscape design^24^.

Different types of organic mulch have proved to be effective for the growth of landscape plants and weed management^25,26^. Yard waste such as wood chips and litter have the potential to rejuvenate poor soils and restore nutrient cycling processes in ornamental landscapes^3^. This further illustrates that organic mulch can be used as an auxiliary for green space construction in urban areas to achieve the goal of water and soil conservation and soil nutrient improvement^27,28^. Although, organic mulch is increasingly used in developing countries, there is a great need to enhance the knowledge and expand the use in urban areas^29^.

Many researchers have studied the effect of organic mulch on improvement of soil conditions and water conservation involving relationships between mulch thickness, structure, ratio of mulch components, soil water content, soil temperature, and generation of runoff. However, organic mulch in combination with heavy rainfall in urban areas have not been extensively investigated. According to the "Technical specifications for the application of organic mulch on greening" formulated by Shanghai City, China, the mulch thickness should be between 2 and 10 cm (https://max.book118.com/html/2019/0816/8041110115002042.shtm). The standard mentions that for specific areas with dry climate or soil susceptible to wind erosion, the thickness of mulch should be appropriately modified to ensure the role of organic cover. However, mulch manufacturers indicate that best cover thickness is usually about 5 cm. However, efficient mulch use in urban areas depends on many factors and different requirements as compared to rural areas.

To improve the knowledge urban mulch use efficiency, for better water and soil conservation in cities, research is needed on mulch application rates when the cost is a limiting factor. In this study, rainfall simulation experiments were performed to systematically analyze the soil and water conservation effects of organic mulch in urban applications under different rainfall intensities and slope conditions. Further, the study explored the above relationships with the aim to investigate the efficiency of mulch cover with different application rates that can be beneficial to the use of organic mulch in cities with heavy summer rainfall such as Beijing. Thus, the study provides evidence for the selection of organic mulch cover thickness in urban areas with heavy rainfall and maximizing economic benefits under the premise of saving costs.

## Materials and methods

### Mulch properties

Organic mulch is inexpensive, easy to acquire, and environmentally friendly, especially when it is already decomposed. In this state, it will add nutrients to the soil and promote soil fertility and productivity^1^. In this experiment, colored organic mulch was produced and handled by a mulch manufacturing company, which processes forest organic waste through collection, crushing, screening, sterilization, decaying, and dyeing. The main ingredients include ground wood residue, wood chips, and straw mixture, with a stable composition. The laying density under natural conditions is 0.16–0.20 kg/L. According to the screening method provided by the national “Standard for Organic Substances for Greening”, the particle size of the organic cover was measured, and then it was divided into four particle size ranges: > 20 mm, 10–20 mm, 5–10 mm, and < 5 mm. The proportion between these ranges was 19.2 ± 5.3%, 43.5 ± 4.4%, 22.7 ± 0.9%, and 14.5 ± 3.3%, respectively. Cinnamon, the typical soil in the rocky mountain area of North China, was collected nearby for use in the rainfall experiments. The soil contains clay (0–2 μm), fine silt (2–20 μm), and coarse silt (20–50 μm), sand (> 50 μm), with proportions 9.5, 57.2, 24.8, and 8.5%, respectively.

### Experiments

**Figure 1:**
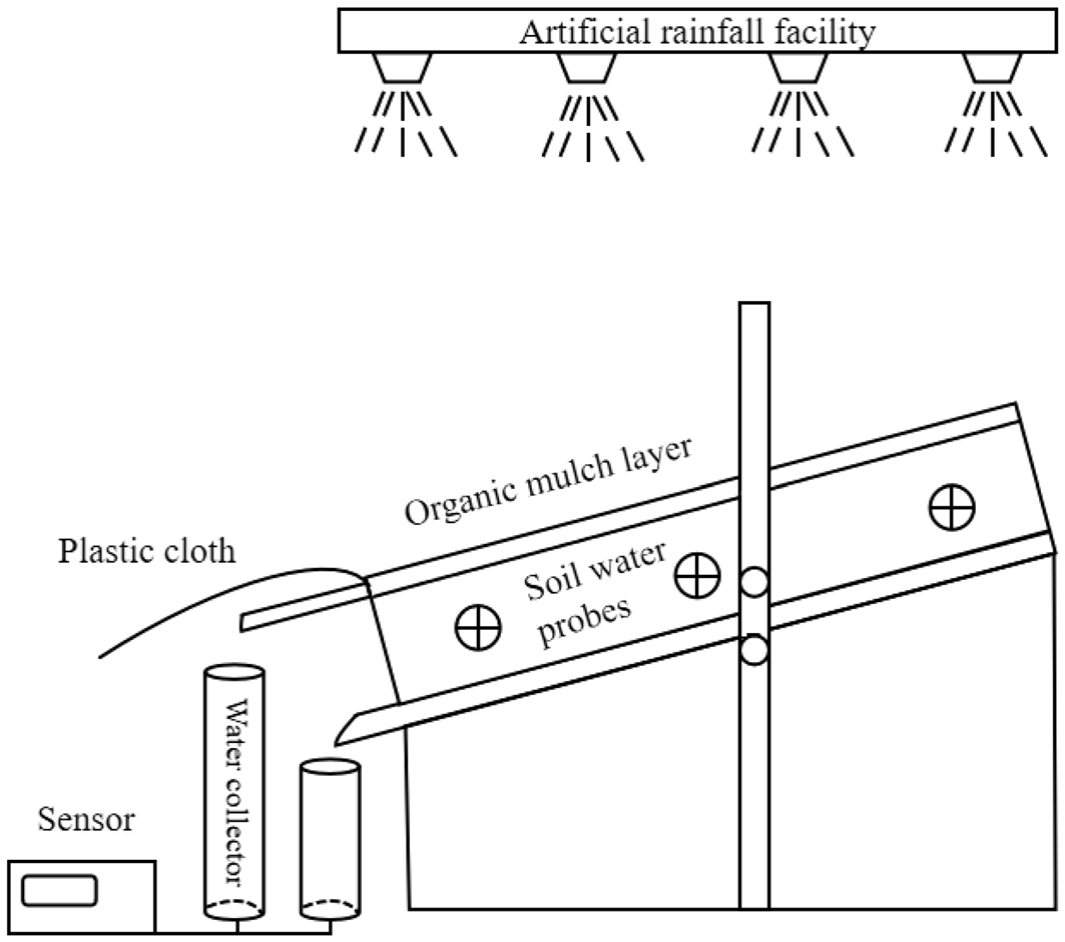
Experimental setup for rainfall and runoff effects of mulch cover.

The experiments were conducted in the rainfall hall of the Capital Forest Ecological Station of the Jiufeng National Forestry Park. The facility is outlined in Fig. 1. It consists of a screen, soil tank, lateral and vertical flow collectors, soil moisture probes, and angle adjustment bracket. The screen has an area of 1.2 m × 0.5 m for holding organic mulch. The size of the soil trough is the same as that of the litter placing sieve, with a depth of 12 cm. In the experiments, the soil depth was equal to that of the tank. The surrounding and bottom of soil tank, as well as the front section of the feeding screen, were all lined with 1.5 cm × 1.5 cm metal mesh to ensure that vertical and horizontal flow were not obstructed and to prevent organic mulch sliding. The lateral and vertical flow collectors measured real-time amount of water by a water pressure sensor (Sino measure, sin-p300) fixed to the bottom, with a range of 0–50 kPa. The pressure sensor was connected to a paperless recorder (Sino measure, sin-r2000) to automatically record time. The upper part was covered with plastic film to prevent rainfall from entering the collection barrels. Soil moisture probes were inserted into the soil at depth of 6 cm in the upper, middle, and lower parts of the tank, respectively, to provide data on changes in soil water content. The experiments provided data readings every 30 s that were recorded. The adjustable bracket was used to adjust the inclination angle of the soil groove. As shown in Fig. 1, the angle adjustment bracket mainly contains an iron rod with a hole. During the testing, another iron rod was inserted into the hole and supported the soil groove to control the angle. The two holes represent 15° and 25° from bottom to top. The size of the base shelf is 1.2 m × 0.5 m × 0.15 m.

The artificial rainfall experiments were performed using the QYJY-203C artificial rainfall simulator. Its effective rainfall height is 12 m, with a variation range of rainfall intensity between 10 and 300 mm/h. The rainfall process is automatically controlled through a computer console, and the uniformity of rainfall, which reflects the unit weight of rainwater in different locations of the distribution of uniformity, is up to 80%. The rainfall experiments were designed to mimic typical extreme rainfall intensity of the Beijing area. According to observations at the Beijing Meteorological Observatory during the past 30 years, maximum hourly rainfall ranged between 20–90 mm^30^. Thus, two intensities, 60 and 90 mm/h were chosen. The simulated rainfall event was set to a one hour duration and observations started from the end of each rainfall simulation. They were halted when the dripping process of water intercepted by the mulch layer ended. The rainfall intensity was calibrated and adjusted before each rainfall^31^.

### Experimental procedure

During the rainfall experiments, three types of water transport were recorded, lateral flow, vertical flow, and mulch interception. Lateral flow includes the water flow generated from surface and interior of mulch layers. Vertical flow refers to water that flows vertically through the pores between the surface and ground cover layer. Using the water balance, the mulch interception was indirectly calculated. As the laboratory experiments were conducted inside a building without wind and sunlight, evaporation was neglected^31^.

**Figure 2:**
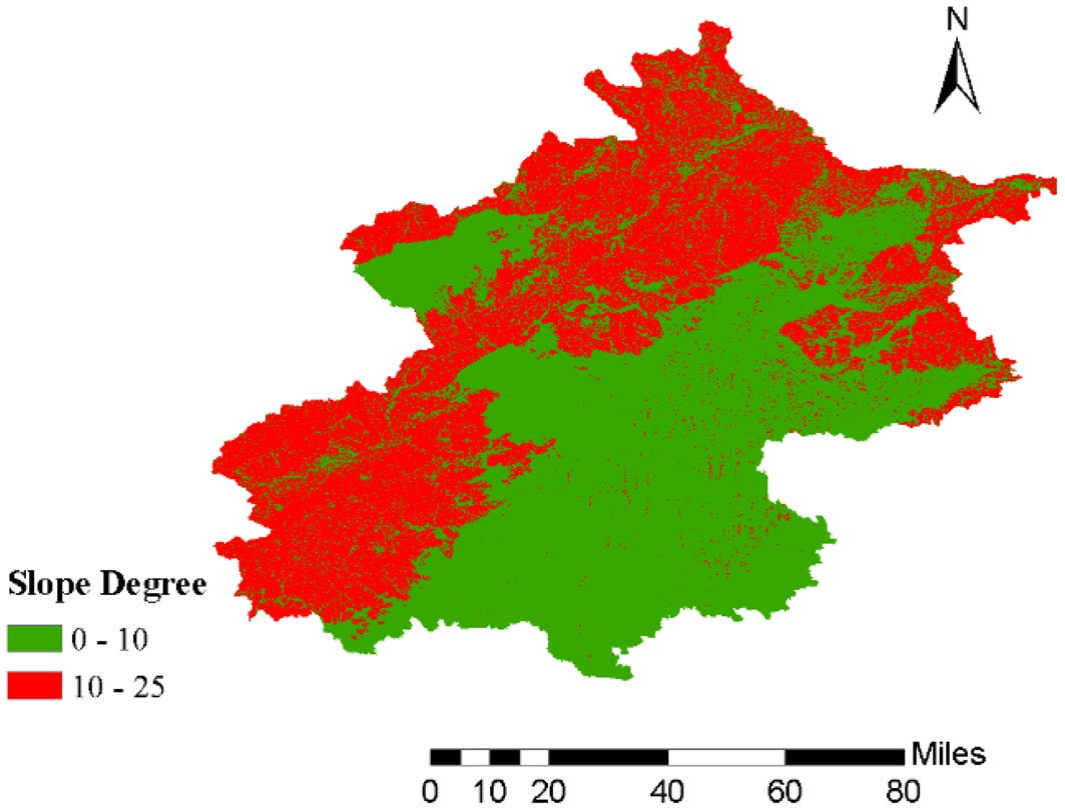
Slope classification in the Beijing area.

Given that the slight move of organic mulch in the process of experiment will exert a certain degree of influence on the measure thickness taking cm as unit, so we chose the average thickness (weight of per unit area) as application rates including 0.25, 0.50, 0.75, and 1.00 kg/m^2^ and a bare soil control group without mulch layer. According to DEM statistics from Geographical Information Monitoring Cloud Platform (http://www.gscloud.cn/) and slope analysis results in Arc GIS 10.2 software, Beijing’s surface slope range is mainly between 0°and 25° (Fig. 2). While the rainfall distribution of Beijing show that abundant and high intensity rainfall events tend to occur in the northwest districts, in which the slope range is focus on 10 ~ 25°according to its DEM data^32^. Hence, two slopes were used for the experiments, 15° and 25°. The experiments included 20 rainfall events.

### Statistical analysis

Five mulch application rates and two slopes were used in the experiments. Pearson’s correlation coefficient (R) was used to calculate the relationships between water holding capacity of the mulch and application thickness with 95% statistical confidence (p < 0.05). Nonlinear regression was conducted to describe the relationship between average runoff generation rate and thickness of mulch. One-way analysis of variance was conducted to explore the relationship between slope and soil water content. The coefficient of determination (R^2^) was used to assess the model fit. Statistical analyses and plots were performed using Excel 2013 and Origin Pro 8, respectively.

## Results

### Effect of organic mulch on soil moisture

**Figure 3:**
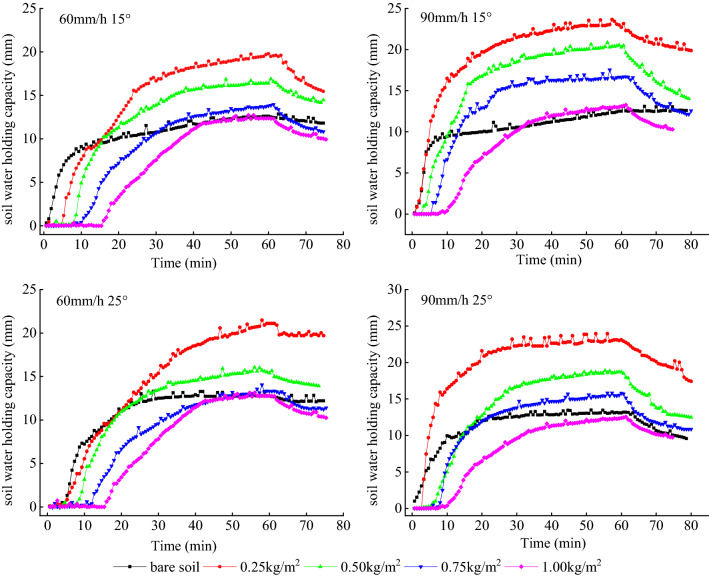
Soil water holding capacity depending on time from start of rainfall.

Figure 3 shows the soil water holding capacity as a function of time for different slopes and rainfall intensities. As seen from the figure, the process of infiltration and soil water content can be divided into four general stages: constant soil moisture (0–10 min), rapid growth of infiltration (10–25 min), slow growth (25–60 min), and rapid decrease of soil moisture at the end of rainfall (60–80 min). The appearance of the first stage demonstrates that the bare soil more effectively infiltrates the rainfall in the early stages of rainfall. However, the overall water retention effect appears largest for the smallest mulch application (0.25 kg/m^2^) (Fig. 3).

The soil water holding capacity under different slope and rainfall intensity decreased significantly with the increase of mulch application. The soil moisture content decreased by 0–13.4 mm with mulch application increase by 0.25 kg/m^2^ for the same rainfall intensity. Compared to bare soil, the varying application rate of mulch exerts varying effect on the soil water holding capacity. The 0.25 and 0.50 kg/m^2^ mulch applications had distinct positive effect on the increase of soil water content, while for the application of 1.00 kg/m^2^, the soil water content tended to be less than for the bare soil. The 0.75 kg/m^2^ application resulted in similar results as for the bare soil. Under same rainfall intensity, soil water content for 0.75 kg/m^2^ mulch application was closer to that of bare soil with the increase of slope. Table 1 shows mean soil water content for the different treatments. The table shows that under the same rainfall intensity, slope does not exert obvious influence on the change of water storage. The ANOVA results showed that at a 95% confidence interval (Table 2), there was no difference between soil moisture for the two slopes. Thus, it appears that slope had no effect on soil moisture.

**Table 1:** Soil water storage under different mulch application and rainfall intensity.

Mulch application (kg/m^2^)	Rainfall intensity (mm/h)	Water storage (mm)			
		60 mm/h		90 mm/h	
	Slope (°)	15°	25°	15°	25°
0		12.6	12.8	12.5	13.1
0.25		19.8	21.1	22.7	23.1
0.50		16.4	15.4	20.3	18.7
0.75		13.8	13.3	16.6	15.8
1.00		12.4	12.8	13.1	12.4

**Table 2:** Variance analysis for different slopes.

Rainfall intensity	Error	SS	df	MS	F	P-value	F crit
60 mm/h	Between-group error	0.016	1	0.016	0.00144	0.971	5.3
	Within-group error	88.9	8	11.1			
	Total	88.9	9				
90 mm/h	Between-group error	0.441	1	0.441	0.0226	0.884	5.3
	Within-group error	156.2	8	19.5			
	Total	156.6	9				

Final soil water storage under different treatment depends mainly on the mulch thickness. The 0.25 kg/m^2^ application worked best to increase the soil water storage. This application resulted in an increase of 51.7–81.6% soil water retention depending on rainfall intensity and slope as compared to the bare soil. For the application of 0.50 and 0.75 kg/m^2^, the soil water storage increased by 20.3–62.4 and 3.9–32.8%, respectively. Thus, organic mulch can effectively increase the soil water content. However, for a large application of mulch it appears that lateral flow increases and less water is stored in the soil. Moreover, large application of mulch decreases the rainwater energy and reduces flow velocity. Thus, less water and soil drainage reduce the amount of water stored in the soil layer.

Soil infiltration rate is an important indicator of soil water conservation capacity, and the larger soil infiltration rate reflects that rainfall is likely to transfer into soil water rather than producing runoff and erosion^26^. Table 3 presents the soil water infiltration rate for different slope, thickness, and rainfall intensity. The infiltration rate of the organic mulch shows a similar trend. Increasing mulch application reduces the infiltration. The average infiltration rate for soil covered by 0.25 kg/m^2^ mulch increased by 29.2–77.4% as compared to the bare soil. For 0.50 and 0.75 kg/m^2^ the same value was 7.1–44.5% and 3.1–17.4%, respectively, and for 1.00 kg/m^2^ it was 0.3–25.7%. However, for a rainfall intensity of 60 mm/h and 15° slope, the soil infiltration rate displayed different trend depending on application. Soil infiltration increased from 0.25 to 0.75 kg/m^2^, and then dropped for 1.00 kg/m^2^ close to the value of the bare soil. Although, the maximum infiltration rate (Cmax) for rainfall intensity of 60 mm/h and the slope 15° reached a peak for 0.75 kg/m^2^, the standard deviation shows that data are highly variable. But for other conditions, the Cmax reduced gradually to that of bare soil from 0.25 to 1.00 kg/m^2^ and reached a maximum for 0.25 kg/m^2^. For the 1.00 kg/m^2^ mulch cover, both maximum and average infiltration rate were close to those of the bare soil. Considering these two indicators, the most efficient mulch cover to improve soil water infiltration rate appears to be about 0.25 kg/m^2^.

**Table 3:** Soil infiltration rate C_max_ of organic mulch for different rainfall intensities (mm/min).

Slope (°)	Thickness (Kg/m^2^)	60 mm/h		90 mm/h	
		Cmax	$\overline{C}$ ± STD	Cmax	$\overline{C}$± STD
15°	0	39.05	30.02 ± 8.89	33.88	26.68 ± 6.11
	0.25	57.09	38.77 ± 18.58	46.31	37.82 ± 10.24
	0.50	56.43	41.19 ± 17.18	46.02	36.31 ± 11.44
	0.75	64.57	44.35 ± 24.38	34.93	28.15 ± 7.93
	1.00	38.17	22.84 ± 14.29	39.78	26.61 ± 13.42
25°	0	39.93	32.48 ± 10.64	40.48	33.89 ± 7.73
	0.25	64.42	44.72 ± 19.46	71.81	59.70 ± 14.94
	0.50	48.19	34.77 ± 15.17	56.67	41.19 ± 16.64
	0.75	42.19	26.58 ± 14.03	47.51	35.00 ± 13.65
	1.00	39.4	23.53 ± 14.76	37.66	25.19 ± 12.71

C_max_ indicates maximum infiltration rate, and $\overline{C}$, STD indicates standard deviation of average infiltration rate for the different experimental conditions.

### Effect of organic mulch on runoff

**Figure 4:**
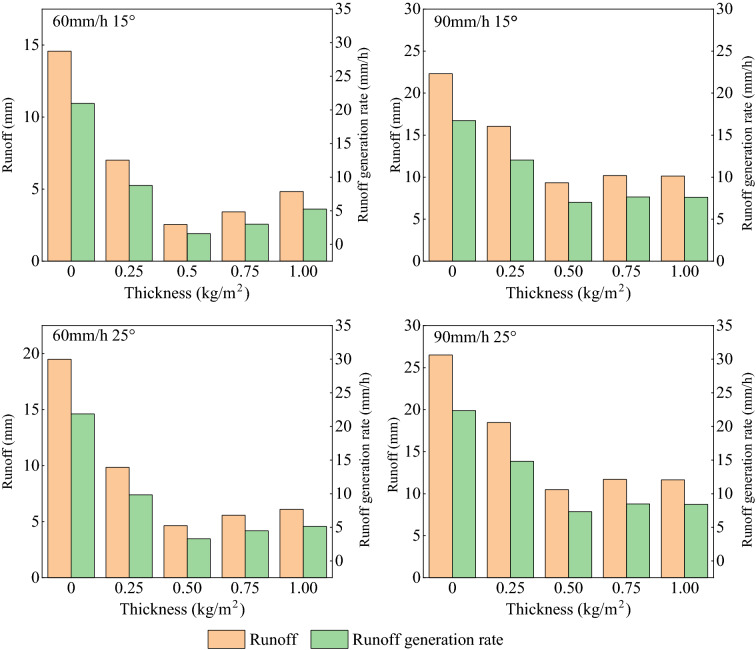
Relationship between mulch application and runoff.

Much research has been performed on organic mulch and effects on runoff, energy of runoff and impeding soil particles, and loss of nutrients^33–35^. Runoff and its generation rate are most common parameters for evaluation of the extent to which runoff causes erosion. Runoff generation rate refers to the flow rate per unit time, which is an important index to describe the flow generation process on slope. The relation between mulch thickness, runoff, and runoff generation rate is shown in Fig. 4. In the experiments, runoff and runoff generation rate for organic mulch were significantly reduced compared to the bare soil. With increasing mulch application, these two parameters showed the same pattern of change. First, there was a decrease and then a slight increase. Both showed a minimum for about 0.50 kg/m^2^ application of mulch implying that this rate is effective in controlling runoff and reducing erosion.

Compared to bare soil, the runoff and runoff generation rate, were in general reduced by 28.1–82.5 and 21.0–82.7%, respectively, as depicted in Fig. 4. The runoff generation rate changed with the variation of application when other variables were kept constant. This means that the mulch application has a certain influence on the average flow generation rate. Correlation analysis showed that the mulch application had effect on the runoff generation rate, as presented in Table 4.

**Table 4:** Significant correlation and regression analysis between application rate (x) and average runoff rate.

Experiment conditions	Regression equation	Correlation coefficient (R^2^)
60 mm/h 15°	y = 13.207–62.882x + 129.629x^2 ^− 84.748x^3^	0.771
60 mm/h 25°	y = 17.565–86.285x + 193.549x^2 ^− 133.803x^3^	0.719
90 mm/h 15°	y = 20.145–59.661x + 125.122x^2 ^− 85.129x^3^	0.569
90 mm/h 25°	y = 23.914–75.297x + 157.773x^2 ^− 106.399x^3^	0.587

**Figure 5:**
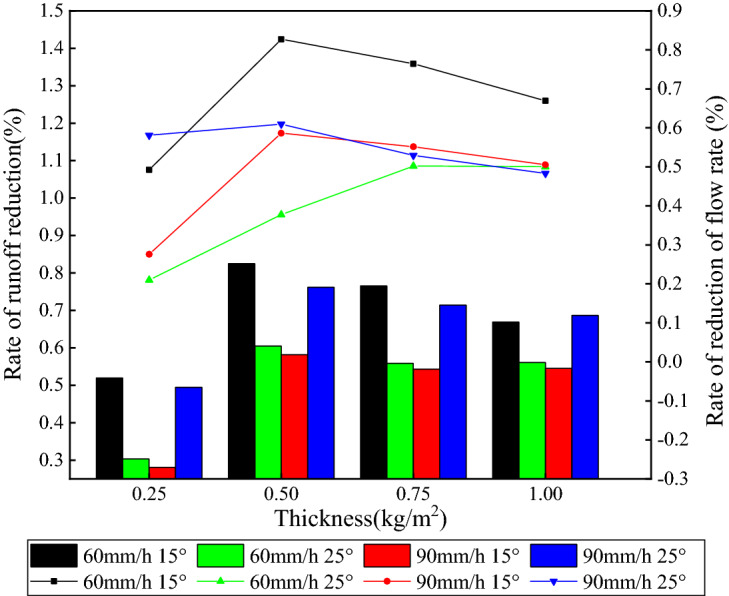
Relationship between application of mulch and runoff reduction.

The runoff reduction rate was generally at maximum for 0.50 kg/m^2^ mulch application. Figure 5 presents the reduction rate of runoff generation, which directly affects the formation of runoff in the process of rainfall and has a corresponding negative correlation.

### Effect of organic mulch on the rainfall distribution process

**Figure 6:**
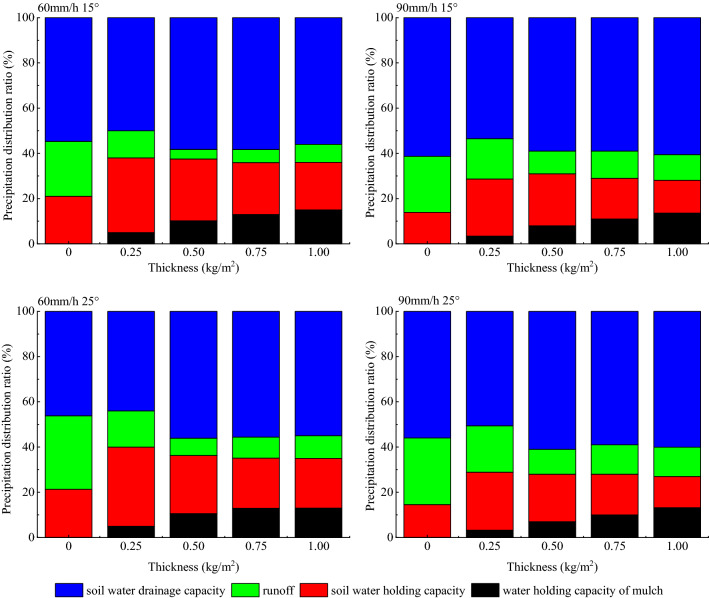
Precipitation distribution into water balance components.

The rainfall-runoff process includes four water balance parts: runoff, soil drainage, soil water, and mulch water. The bare soil water balance includes runoff, soil moisture, and soil drainage, which accounted for 24.3 ~ 32.5, 13.9 ~ 21.3, 46.2 ~ 61.3 of total rainfall, respectively (Fig. 6).

**Figure 7:**
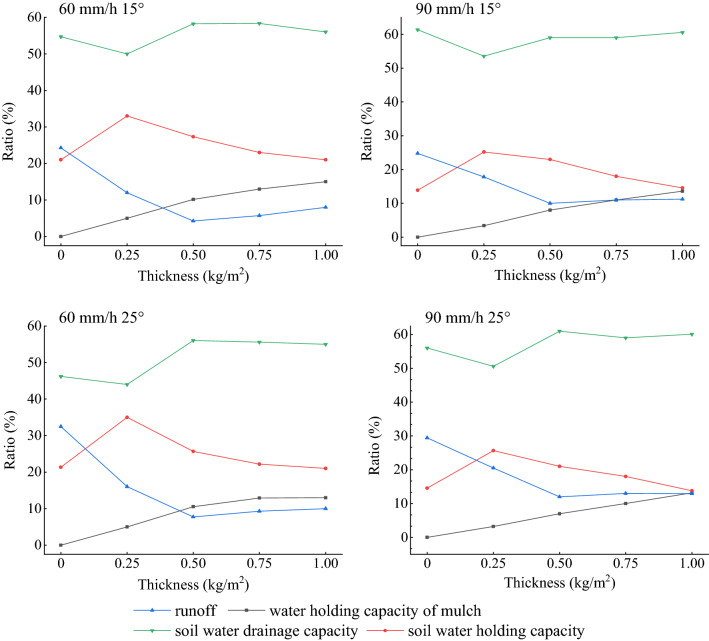
Relationship between water balances from rainfall input depending on mulch application.

The soil drainage displayed a common trend for all variables as shown Fig. 7. This means that an increase of mulch application slightly decreased the soil drainage. It represented 56% ~ 60% of the total precipitation input, which was slightly higher than that of the bare soil. For a rainfall intensity of 90 mm/h, with application increase from 0 to 1.00 kg/m^2^, soil drainage for the two slopes declined to 48.2 and 45.5 mm from 55.2 and 50.4 mm, respectively, and eventually, increased to 54.5 mm and 54.1 mm, respectively.

The soil water content showed an opposite behavior with the soil drainage. Its ratio increased from 11.1 to 13.7% and then reduced as for the bare soil and increasing application. Its proportion accounted for 13.8 ~ 35%. Meanwhile, the mulch water content showed positive correlation with application, with a 3.2% average increase. The Pearson correlation indicates that mulch water content has a significant positive relationship with application (Table 5, *P* < 0.05).

**Table 5:** Pearson correlation coefficient R between water holding of organic mulch and application of mulch with slope.

Experiment variables	Thickness—slope			
	60 mm/h 15°	60 mm/h 25°	90 mm/h 15°	90 mm/h 25°
Water holding content of mulch	0.984**	0.997**	0.956*	0.999**

Two-tail test of significance was used and significant correlations (p < 0.01) are labeled with **, significant correlations (p < 0.05) are labeled with *.

The change of runoff, with the increase of mulch application, first underwent a sharp drop that was followed by a slow rise. But the runoff for the organic mulch covered plots declined by 8.9–24.8% for all slopes when compared to the bare soil. A thickness of 0.50 kg/m^2^ appears more effective as compared to other thicknesses.

The soil drainage accounted for the largest portion, 46.2–61.3% of the water balance. Second in size was the soil water content, which continuously declined with 0.25 to 1.00 kg/m^2^ mulch application. The amount of runoff depends on thickness but decreased by 14.1–24.8% as compared with that of the bare soil. The runoff amount had an opposite behavior with soil water content, causing the two lines (Fig. 7) to intersect at a point between 0 to 0.25 kg/m^2^. The runoff amount was smaller than the soil water content for a thickness of 0.25 kg/m^2^.

The relationship between runoff and mulch water content changed depending on slope and application. The water content increased with increasing mulch thickness. And runoff changed by firstly a decrease and then an increase. At a rainfall intensity of 60 mm/h, runoff amount and soil water content were equal at an application range of 0.25–0.50 kg/m^2^, and at the rainfall intensity of 90 mm/h, the two variables were equal at an application of 0.75–1.00 kg/m^2^. In addition, before the equal range, runoff amount was larger than the mulch water content, and the mulch water content was larger than runoff amount after this equal range for all experiments.

Overall, when the mulch application increased, the difference between soil water drainage and soil water content for the bare soil and the 1.00 kg/m^2^ application was small for a slope of 15°. It only differed by 0.5 and 1.0% for the two rainfall intensities (60 and 90 mm/h), respectively. Table 6 refers to the precipitation distribution ratio under different consitions and it shows the increase of mulch water content came mainly from runoff, with a rate of 92.0 and 100% for 60 and 90 mm/h, respectively. For a slope of 25°, the runoff water turned into mulch water content and soil drainage. At a rainfall intensity of 60 mm/h and a slope of 25°, runoff into mulch moisture, soil drainage, and soil water content was 57.8, 2.9, and 39.2%, respectively. For a rainfall intensity of 90 mm/h and slope 25° runoff turning into mulch moisture content and soil water drainage was 76.3 and 23.7%, respectively.

**Table 6:** Precipitation distribution ratio under different conditions.

Rainfall intensity-slope	Thickness (kg/m^2^)	Water holding capacity of mulch	Soil water holding capacity	Runoff	Soil water drainage capacity
60 mm/h 15°	0	0	21	24.3	54.7
	0.25	5	33	12	50
	0.5	10.16	27.33	4.26	58.25
	0.75	12.98	23	5.7	58.32
	1	15	21	8	56
60 mm/h 25°	0	0	21.33	32.49	46.18
	0.25	5	35	16	44
	0.5	10.55	25.67	7.73	56.05
	0.75	12.93	22.18	9.28	55.61
	1	13	22	10	55
90 mm/h 15°	0	0	13.89	24.79	61.32
	0.25	3.42	25.23	17.83	53.52
	0.5	8	23	10	59
	0.75	11	18	11	59
	1	13.6	14.56	11.26	60.58
90 mm/h 25°	0	0	14.56	29.44	56
	0.25	3.23	25.67	20.51	50.59
	0.5	7	21	11	61
	0.75	10	18	13	59
	1	13.2	13.78	12.93	60.09

The partitioning of runoff into different water balance components is a dynamic process. When the mulch application increased to 0.50 kg/m^2^, both soil water content and runoff declined and were converted into mulch water and drainage. The distribution for the application of 0.75 and 1.00 kg/m^2^ meant a decrease of soil water and increase of mulch water and runoff.

## Discussion

Different types of organic mulch have varying performance under different conditions^36,37^. Shojaei et al. performed experiments to determine optimal mulch composition to minimize effect of wind erosion. They found that a mulch thickness of 1.5 mm consisting of straw (5.30 g/m^2^), manure (15.66 g/m^2^), biosolids (16.08 g/m^2^), and other black covering material (12.46 g/m^2^), was optimal for controlling wind erosion^5^. Our study focused on the effects of organic mulch thickness on rainfall input partitioning depending on slope and rainfall intensity for urban conditions. The components of the manufactured mixed organic mulch material used in the experiments are biologically stable with a determined particle size distribution. Their physicochemical properties need to be further explored and determined in detail. However, due to that the mixed material composition is stable and homogeneous, quantitative differences of soil and water contents are reliable.

Organic mulch has advantages such as low cost, easy to acquire, environmentally friendly, and constituting soil nutrients. Organic mulch consisting of single and mixed components was used in this study that explored the function of soil and water conservation. Previous research has often been based on comparison of different types of organic mulch. This has included studies of crop yield, soil water use efficiency, soil moisture increase, variation of soil organic matter after mulching, and amount of weed. The organic mulch used in our study, was processed in factory and thus, ensured homogeneous and standard material composition. However, in practical application of organic mulch, especially when using mulch from nut orchards, local material can be used to achieve the purpose of water conservation and fertilizer at a low cost. For chestnut plantations, weed and litter layers are cleared before the chestnuts are harvested. After the harvest, bark, dead branches, and weed are burned or buried in the process of clearing to eliminate surviving bacteria and pest^38^. The understory of Chinese chestnut forest is bare and lack of litter and weed cover, resulting in soil and water loss, which affects the yield and the growth quality of seedlings in a negative way^39^. Studies have shown that covering with grass and wheat straw can effectively improve the overall quality of Chinese chestnut about 10%^40^. In addition, through the understanding of the market price, plastic mulching film with the size of 1.2 m*0.5 m covering cost 0.04 yuan, and the organic mulching layer with an application rate of 0.25 kg/m^2^ costs 1.2 yuan. Plastic film due to environmental protection has been gradually phased out^41^, although the cost of organic mulch is higher, it is very low compared with the application of advanced technology treatment such as degradable mulch that may cost 0.84 yuan. In general, organic mulch is a useful method to prevent loss of water and soil and increase yield of orchards.

Research shows that it is better to use chopped biomass of semi-hard woody perennial plants instead of crop residues to cover the soil surface^12^. Research on utilization of agricultural and forest waste, has shown that the current production of wood processing wastes and agricultural straw in China corresponded to about 500 million ton of standard coal, which is expected to reach 900 million ton by 2020^42^. In future, resources will be integrated and diversified to achieve sustainable development and promote the utilization of agricultural and forestry wastes. Using forestry waste to produce organic mulch or degradable mulch is an important way to integrate resources.

Mulch plays an effective role in conserving water and soil. An 8-year study performed by Li et al. illustrated that long-term mulching treatment with straw did not improve soil water holding properties for a soil depth of 0–30 cm^43^. They found that among all treatments (1, 2, 4, 8, and 12 metric ton/ha), the increase of soil water and yield were greatest for the 12-metric ton/ha mulch but benefits at this rate were not much greater than those of 8 tons/ha^44^. In our experiments, soil water content under mulch cover increased for all mulch applications (0–1.00 kg/m^2^). However, 0.25–0.50 kg/m^2^ mulch application maximizes the positive effects on soil water and runoff. The most likely reason is that short-term mulching treatment does not change soil water movement and influence deeper layers of soil^45^. A similar long-term study on wood chip mulch thickness effects on soil water and plant growth was conducted by van Donk et al.^26^. They studied soil water content for a 0–0.61 m soil depth and three mulch thicknesses (2.5, 5.0, and 10.0 cm) under natural rainfall conditions. A mulch cover thickness of 5 cm gave greater soil water content than other thicknesses^26^. The discrepancy between these two results may stem from the effect of the longer mulch treatment time.

The simulation experiment was conducted for a Beijing climate with characteristics of rainy season rainfall intensity. By consideration of soil water content, runoff, runoff rate, and other indicators, an optimum application range of 0.25–0.50 kg/m^2^ was obtained under two rainfall intensities and slopes. Although, results were variable, consistent results displayed that a mulch thickness range of 0.25–0.50 kg/m^2^ was most efficient. In this experiment, the soil water content was approximately constant during the first 10 min after onset of rainfall due to that the cumulative rainfall was completely intercepted by the mulching layer. This phenomenon is unfavorable regarding the growth of trees and other vegetation in areas with heavy rainfall of a duration of less than 10 min. According to climate data of Beijing in recent ten years, heavy rainfall events has a low occurrence probability but a long duration of 7–8 h/year meaning that each rainfall event lasts more than 10 min^46,47^. This means that organic mulch can be used in both urban and rural areas, and at the same time, the application rate can be roughly selected by referring to our experimental results. However, our indoor tests did not simulate effects of rainfall intensity variation. In the future, variable rainfall intensity can be used for more close field-scale conditions^48^.

Previous research has shown that mulching practice has effect on sediment reduction, and generally (1) reducing raindrop impact, (2) increasing water infiltration, (3) increasing surface storage, (4) decreasing runoff velocity, (5) improving soil structure and porosity, and (6) improving the biological activity in the soil^49^. Smets et al. (2008) compared 41 studies to find spatial scale effects on the effectiveness of organic mulch in reducing soil erosion by water, and concluded that for varying scales, experimental variables such as slope gradient, experiment duration, soil texture, mulch type and length of slope) exerted different degrees of influence on the mulch function on reducing sediment loss and soil erosion. Our laboratory experiments, carried out on slots of 1.2 m × 0.5 m, can be said to belong to a microplot/mesoplot scale. Out of the 41 studies mentioned above, splash erosion, interrill erosion, and rill erosion occurred, but local circumstances exert varying effects on the different processes. Thus, laboratory experiments are needed to further explore detailed relationships of soil conservation function and experimental variables (mulch type, slope, soil texture, and slope length).

## Conclusion

In the present study, artificial rainfall experiments were applied to determine the water and soil conservation function of varying application rates of mixed organic mulching under simulated urban conditions of heavy rainfall. Soil water content processes in mulch covered soil consisted of four stages: constant soil moisture (0–10 min), rapid growth of infiltration (10–25 min), slow growth (25–60 min), and rapid decrease of soil moisture at the end of rainfall (60–80 min). Comparing the mulching treatment to the bare soil, soil water content increased, and the amount of water conserved in the soil decreased with the increase of mulch application. The 0.25 kg/m^2^ application worked best to increase the soil water storage in the range 51.7–81.6%. The generation of runoff was least for the 0.50 kg/m^2^ mulch application, causing a decrease of 58–83%. The applied rainfall was distributed into four water balance parts: soil drainage, soil water content, mulch water content, and runoff. Among these, the largest was soil drainage > soil water content. Only the mulch water content was positively correlated with mulch application. The relationship between mulch application and investigated hydrological variables, a mulch cover of 0.25–0.50 kg/m^2^ appeared to be optimal.

## Data Availability

All data generated or analyzed during this study are included in this published article (and its Supplementary information file).
